# Fatty Liver Index vs. Biochemical–Anthropometric Indices: Diagnosing Metabolic Dysfunction-Associated Steatotic Liver Disease with Non-Invasive Tools

**DOI:** 10.3390/diagnostics15050565

**Published:** 2025-02-26

**Authors:** Selim Demirci, Semih Sezer

**Affiliations:** Department of Gastroenterology, Dr. Abdurrahman Yurtaslan Oncology Training and Research Hospital, 06200 Ankara, Turkey; ssezer1970@hotmail.com

**Keywords:** anthropometry, metabolic dysfunction-associated steatotic liver disease (MASLD), non-invasive diagnosis, triglyceride–glucose index, fatty liver index

## Abstract

**Background/Objective:** Metabolic dysfunction-associated steatotic liver disease (MASLD) has emerged as a significant global burden, attributed to its increasing prevalence and strong correlation with metabolic syndrome and related conditions. Timely diagnosis and intervention are essential for minimizing the impact of MASLD. This study sought to analyze the efficacy of advanced anthropometric indices and non-invasive steatosis markers in diagnosing MASLD. **Methods:** This cross-sectional retrospective study evaluated the data from 578 Turkish patients admitted to our gastroenterology clinic. MASLD was diagnosed based on internationally recognized criteria. The evaluated parameters included body mass index (BMI); waist–hip ratio (WHR); waist–height ratio (WHtR); body roundness index (BRI); conicity index (CI); a body shape index (ABSI); visceral adiposity index (VAI); abdominal volume index (AVI); lipid accumulation product (LAP); fatty liver index (FLI); hepatic steatosis index (HSI); and triglyceride–glucose index (TyG) and its variants TyG–waist circumference(WC) and TyG–BMI. **Results:** Among 215 men, 103 (56.9%) met the criteria for MASLD, while 260 out of 363 women (65.5%) fulfilled the criteria. In the receiver operating characteristic (ROC) analysis for identifying MASLD, TyG–WC (0.826), TyG–BMI (0.820), and FLI (0.830) achieved the highest area under the curve (AUC) values, with statistically significant differences observed in their pairwise comparisons against the other parameters. **Conclusions:** TyG–WC and TyG–BMI are comparable to FLI in terms of simplicity of calculation and superior diagnostic accuracy, making them valuable non-invasive alternatives for MASLD screening and diagnosis.

## 1. Introduction

Non-alcoholic fatty liver disease (NAFLD) describes a variety of disorders characterized by the atypical buildup of fat within hepatic cells [1]. Globally, NAFLD stands out as the leading cause of liver conditions, impacting approximately 25% of the population [2]. NAFLD often occurs alongside metabolic syndrome-related conditions such as diabetes, impaired fasting plasma glucose (FPG), hypertension, obesity, dyslipidemia, and cardiovascular diseases [3]. The term “metabolic dysfunction-associated fatty liver disease” (MAFLD) emerged in 2019 as a proposed replacement for “NAFLD”, addressing the inadequacy of the term “non-alcoholic” in describing the disease’s metabolic associations [4]. MAFLD emphasizes its connection to metabolic risk factors and is recognized as a positive diagnosis that incorporates other chronic liver diseases. It is categorized into three subgroups based on diabetes, obesity, and other metabolic components. However, a notable limitation of MAFLD is its inclusion of not only metabolic dysfunction but also factors like alcohol consumption and other liver diseases, which complicates the understanding of disease pathogenesis and the identification of homogeneous patient groups. To address these challenges, an updated term, “metabolic dysfunction-associated steatotic liver disease” (MASLD), was suggested in 2023 as an updated classification for steatotic liver disease (SLD) [5]. This new term aims to replace earlier terminologies that included potentially stigmatizing labels and to provide a more comprehensive framework for describing the range of fatty liver diseases. The pathogenesis of MASLD remains incompletely understood, driven by a multifaceted interplay of genetic predisposition, intrahepatic lipid deposition, impaired insulin metabolism, and chronic inflammatory mechanisms [6]. MASLD includes a variety of clinical conditions, beginning with simple steatosis and progressing to steatohepatitis, which can subsequently advance to severe fibrosis, cirrhosis, and eventually hepatocellular carcinoma in later stages [7]. With its prevalence increasing due to lifestyle changes and rising obesity rates, MASLD poses a significant risk not only for liver-related health complications but also for lifestyle-associated disorders such as cardiovascular conditions, type 2 diabetes, and chronic kidney disease (CKD) [8,9]. Therefore, the early identification of MASLD is crucial, not only for preventing liver-related complications but also for reducing the risk of associated conditions. However, early-stage MASLD often progresses silently, as most patients exhibit no symptoms, creating a substantial barrier to its early detection and preventive strategies [10].

Unlike NAFLD, which is diagnosed by excluding other chronic liver conditions and alcohol use, MASLD is identified based on specific positive criteria. MASLD can be confirmed when hepatic steatosis is present alongside at least one cardiometabolic risk factor [11]. While liver biopsy continues to be the definitive method for diagnosing fatty liver disease (FLD), its invasiveness, limited patient acceptance, high cost, and sampling variability render it impractical for routine practice. Imaging modalities used to detect hepatic steatosis are often dependent on specialized equipment and personnel, leading to accessibility challenges and additional cost burdens. Furthermore, the diagnosis of MASLD is not limited to the identification of hepatic steatosis alone but also requires the presence of accompanying risk factors, making it less practical for use in large-scale epidemiological studies. Therefore, there is a pressing need to develop a simple tool for identifying MASLD. The liver is a key organ in regulating both glucose and lipid metabolism. In patients with hepatic steatosis, the inability to suppress lipolysis regulated by insulin results in increased concentrations of free fatty acids (FFAs), leading to their excessive release into the bloodstream [12]. This increased influx of FFAs promotes the production of very-low-density lipoprotein (VLDL), hindering the efficient clearance of triacylglycerol and contributing to hypertriglyceridemia and insulin receptor dysfunction [13]. Building on these biochemical pathways and earlier methods, specialized scoring systems integrating biochemical and clinical factors have been created to identify hepatic steatosis and assess MASLD likelihood. The fatty liver index (FLI) is a commonly utilized diagnostic panel that includes gamma-glutamyl transferase (GGT), triglyceride (TG), BMI, and WC. Similarly, the hepatic steatosis index (HSI) incorporates diabetes status, BMI, and the alanine aminotransferase (ALT)/aspartate aminotransferase (AST) ratio [14]. Nonetheless, the computation for both indices remains intricate, requiring the integration of numerous parameters and variables. Given the global prevalence of the disease, accessible and straightforward methods for early diagnosis and timely intervention are crucial for mitigating its impact.

One such non-invasive quantitative measurement tool available to clinicians is anthropometry. It encompasses the measurement of physical dimensions and body composition to assess human physical characteristics. These measurements include height (Ht), body weight (BW), hip circumference (HC), waist circumference (WC), and skinfold thickness measurements [15]. In particular, the body mass index (BMI), calculated using Ht and BW, is a widely utilized index for classifying obesity. However, in addition to not providing information about fat distribution in the body, BMI also has limitations in predicting metabolic disorders [16]. Therefore, beyond BMI, there has been a need for additional anthropometric measurements, indices, and methods. Therefore, advanced anthropometric indices such as body roundness index (BRI), conicity index (CI), a body shape index (ABSI), visceral adiposity index (VAI), abdominal volume index (AVI), lipid accumulation product (LAP), and triglyceride–glucose index (TyG), along with TyG–WC and TyG–BMI, have been developed using basic measurements such as Ht, BW, HC, WC, and certain biochemical parameters. Extensive research has explored the use of these indices across a range of cardiometabolic disorders, including metabolic syndrome [17], diabetes [18], cardiovascular diseases [19], sarcopenia [20], and NAFLD [21].

Given the established utility of indices integrating anthropometric and biochemical parameters in assessing various metabolic disorders, we hypothesize that these indices could function as effective tools for identifying MASLD. Therefore, this study is designed to evaluate the potential of advanced anthropometric indices, derived from key measurement and biochemical parameters, in identifying MASLD, thereby offering a practical and accessible approach for early diagnosis and clinical assessment.

## 2. Materials and Methods

### 2.1. Study Population

A retrospective cross-sectional study was performed by reviewing consecutive patient records from the gastroenterology outpatient clinic at Abdurrahman Yurtaslan Oncology Training and Research Hospital, covering the period from December 2022 to October 2024. Patients over the age of 18 with abdominal ultrasonography results and recorded data necessary for diagnosing MASLD were included in the study. Patients who were pregnant; consumed alcohol exceeding 140 g per week for women or 210 g per week for men; had a history of Wilson’s disease or drug-induced liver disease; were taking medications known to influence liver function (e.g., corticosteroids or hepatotoxic drugs); had congenital metabolic disorders, HCV or HIV infection, and/or malnutrition; or had undergone abdominal liposuction were excluded from this study. This study was formally authorized by the Hospital Ethics Committee (approval No. 2024-11/170) and conducted while respecting the ethical stipulations of the Declaration of Helsinki. Given its retrospective design, obtaining informed consent was deemed unnecessary.

### 2.2. Data Acquisition and Anthropometric Measurements

Demographic data, including age, educational level, exercise habits, and smoking status, were retrieved from system records. Participants underwent blood sampling after fasting for at least eight hours. Liver function parameters, including AST, ALT, and GGT, as well as TG, glucose, and high-density lipoprotein cholesterol (HDL-C) levels, were documented. For all participants, systolic blood pressure (SBP) and diastolic blood pressure (DBP) were recorded in a seated posture following an adequate period of rest. BW (kg) and Ht (cm) were recorded following standardized procedures, with lightweight attire and no footwear. WC was determined by measuring halfway between the iliac crest and the lower rib, whereas HC was taken by wrapping a tape measure around the broadest part of the hips and buttocks, ensuring the tape measure stayed level with the ground. All parameters used for the calculation of non-invasive indices were extracted from electronic medical records of routine clinical evaluations, including anthropometric measurementsand laboratory test results.

### 2.3. Calculation of Anthropometric, Biochemical–Anthropometric, and Non-Invasive Steatosis Indices

Building on prior research [22,23,24], we employed established formulas to calculate the values of various indices, involving waist–hip ratio (WHR), waist–height ratio (WHtR), BRI, CI, ABSI, VAI, AVI, LAP, TyG, TyG–WC, and TyG–BMI. Additionally, FLI and HSI, two of the most commonly used non-invasive steatosis indices, were also calculated.The specific formulas used for these calculations are detailed in Table 1.

### 2.4. MASLD Diagnosis

The determination of hepatic steatosis via ultrasound is based on specific findings, including liver-to-kidney echo contrast, increased brightness of the liver parenchyma, attenuation of the ultrasound signal at greater depths, and blurring of vascular structures. MASLD is identified through hepatic steatosis combined with one or more of the following criteria [5]: (1) a BMI equal to or exceeding 25 kg/m^2^, or a WC of at least 94 cm for men and 80 cm for women; (2) an FPG of ≥100 mg/dL, a history of type 2 diabetes, or its active treatment; (3) a blood pressure of at least 130/85 mmHg or ongoing antihypertensive treatments; (4) plasma TG levels of ≥150 mg/dL or treatment with triglyceride-reducing therapies; and (5) plasma HDL-C levels below 50 mg/dL and 40 mg/dL in women and men, respectively, or current use of lipid-lowering therapies.

### 2.5. Statistical Analyses

Statistical analyses were performed in SPSS version 27 (SPSS Inc., Chicago, IL, USA) and MedCalc Statistical Software version 23.1.3 (MedCalc Software, Ostend, Belgium). Continuous variables were presented as mean ± standard deviation, with normality tested for each parameter. Categorical data were examined using the chi-squared test, while continuous variables were analyzed using either the Mann–Whitney U test or Student’s *t*-test based on their distribution. The diagnostic performance for MASLD was analyzed via a receiver operating characteristic (ROC) analysis, where the area under the curve (AUC) values were computed and statistically compared via the DeLong method. The maximum Youden index was employed to identify optimal cutoff points for each index. To evaluate the odds ratios (ORs) and their 95% confidence intervals (CIs) for MASLD, a logistic regression analysis was performed.

## 3. Results

### 3.1. Characteristics of the Study Population

**Figure 1:**
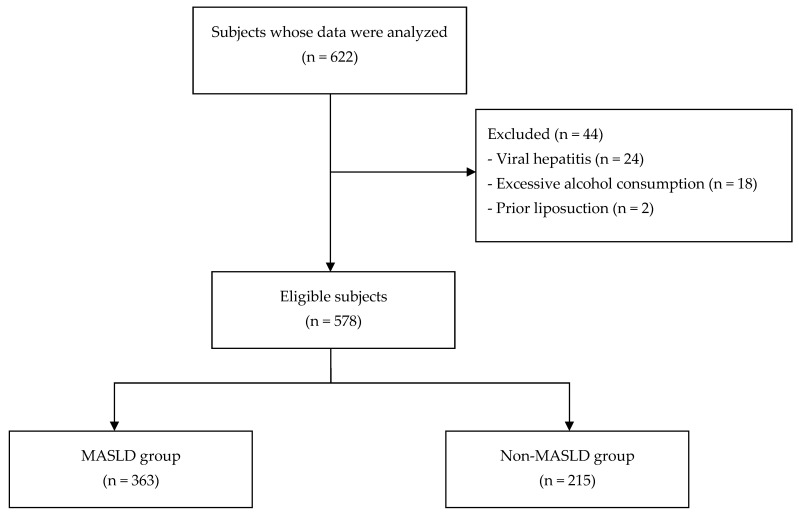
Flowchart of this study. MASLD: metabolic dysfunction-associated steatotic liver disease.

This study involved the evaluation and analysis of data from 578 patients. Comprehensive demographic and anthropometric details are presented in Table 2. Among the 578 participants, 103 out of 181 men (56.9%) and 260 out of 397 women (65.4%) met the criteria for MASLD (Figure 1). No notable distinctions were found between the groups regarding age, smoking status, educational level, or exercise habits for either gender (*p* > 0.05). Except for ABSI in males, all anthropometric measurements were markedly elevated in the MASLD group compared with those in the non-MASLD group across both genders (*p* < 0.05). Moreover, both the FLI and HSI levels were notably elevated in the MASLD group for both genders (*p* < 0.05).

### 3.2. A Comparative Analysis of MASLD Criteria Between Men and Women

Because the diagnosis of MASLD is based on hepatic steatosis accompanied by at least one criterion of metabolic dysfunction, the specific metabolic dysfunction criteria met by men and women were compared and are detailed in Table 3. The HDL-related criterion and the WC criterion were significantly more prevalent among women compared to men (*p* < 0.05), whereas all other criteria showed no statistically significant differences (*p* > 0.05).

### 3.3. Performance Evaluation and Pairwise Comparison of Anthropometric, Biochemical–Anthropometric, and Non-Invasive Steatosis Indices in the Diagnosis of MASLD

**Figure 2:**
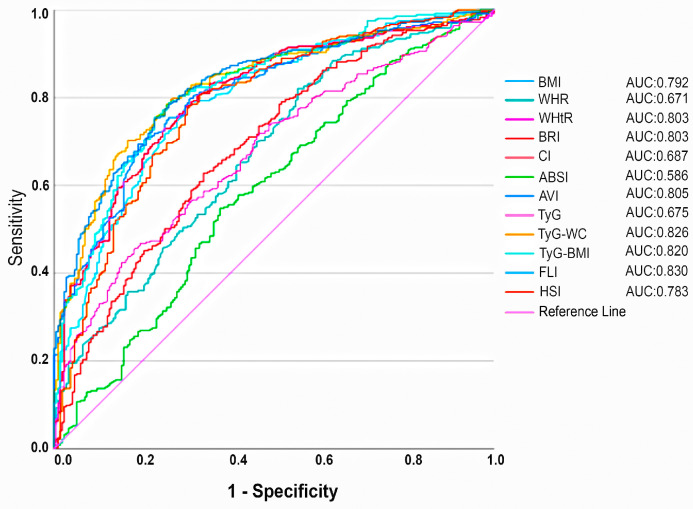
Receiver operating characteristic (ROC) curves of each parameter for diagnosing MASLD. AUC, area under curve; BMI, body mass index; WHR, waist–hip ratio; WHtR, waist–height ratio; BRI, body roundness index; CI, conicity index; ABSI, a body shape index; AVI, abdominal volume index; TyG, triglyceride–glucose index; WC, waist circumference; FLI, fatty liver index; HSI, hepatic steatosis index.

Table 4 presents the ROC analysis conducted to evaluate the diagnostic performance of various indices for MASLD across all cases, alongside the DeLong test results, to assess the significance of differences between the AUC values of these indices (Figure 2). TyG–WC, TyG–BMI, and FLI exhibited strong diagnostic accuracy for MASLD, with all indices achieving AUC values exceeding 0.8.With a cutoff value of 41.21, FLI identified MASLD with a sensitivity of 0.78 and a specificity of 0.74 (AUC: 0.830; 95% CI: 0.797–0.863). While FLI had the highest AUC according to the ROC analysis, its advantage over TyG–WC (AUC: 0.830 vs. 0.826, *p* > 0.643) and TyG–BMI (AUC: 0.830 vs. 0.820, *p* > 0.283) was not statistically significant.

### 3.4. Gender-Based Analysis of the Diagnostic Value of Parameters for Steatosis in Detecting MASLD

**Figure 3:**
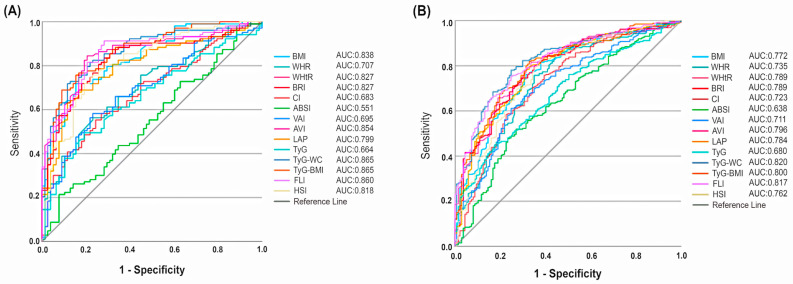
The results of ROC curve analysis for men (**A**) and women (**B**). AUC, area under curve; BMI, body mass index; WHR, waist–hip ratio; WHtR, waist–height ratio; BRI, body roundness index; CI, conicity index; ABSI, a body shape index; VAI, visceral adiposity index; AVI, abdominal volume index; LAP, lipid accumulation product; TyG, triglyceride–glucose index; WC, waist circumference; FLI, fatty liver index; HSI, hepatic steatosis index.

A gender-based analysis of the diagnostic performance of anthropometric, biochemical–anthropometric, and non-invasive steatosis indices in detecting MASLD, assessed through ROC analysis (Figure 3), is presented in Table 5. In men, BMI, WHtR, BRI, AVI, TyG–WC, TyG–BMI, FLI, and HSI, and in women, TyG–WC, TyG–BMI, and FLI emerged as the most effective indices, all with AUC values exceeding 0.8, reflecting strong diagnostic performance.In both genders, the AUC values for TyG–WC and TyG–BMI were found to be higher than those of traditional markers, including FLI and HSI.In men, TyG–WC and TyG–BMI demonstrated the greatest AUC values (both AUC: 0.865, *p* < 0.001) among all indices, whereas in women, TyG–WC achieved the highest AUC value (AUC: 0.820, *p* < 0.001).

### 3.5. Logistic Regression Model

TyG–WC, TyG–BMI, and FLI were evaluated for their relationship with MASLD by categorizing these variables into quartiles, using the first as the baseline reference (Table 6). The findings indicated a robust positive relationship between increasing parameter quartiles and MASLD prevalence, which held statistical significance even after accounting for potential confounding variables (*p* for all <0.001).

## 4. Discussion

This retrospective cross-sectional study evaluated various parameters to assess their effectiveness in diagnosing MASLD. MASLD represents a newly introduced classification, and as far as we are aware, our study is among the limited number of investigations utilizing anthropometric and biochemical–anthropometric indices for the diagnosis of MASLD. Our study demonstrated that TyG–WC and TyG–BMI exhibit comparable diagnostic accuracy to FLI for MASLD diagnosis, reinforcing their utility as practical, non-invasive tools for clinical settings. Despite its increasing global burden, MASLD still lacks approved pharmacological treatments, making lifestyle modifications the cornerstone of prevention and management. Given its rising prevalence and early onset, timely diagnosis should be a clinical priority.

Certain anthropometric indices and lipid parameters have been established as straightforward and effective tools for identifying metabolic syndrome in clinical settings. Scientific findings highlight that BMI and WC show a clear correlation with metabolic syndrome and insulin resistance (IR), as well as a distinct association with hepatic steatosis and steatohepatitis [25]. Moreover, TyG, originally formulated by Simental and colleagues, offers a straightforward yet effective mathematical model for evaluating IR. The computation of TyG is based on parameters such as TG and FPG, which are also utilized in the diagnosis of MASLD, making it particularly suitable for population-based studies. Our findings indicated that TyG had a relatively lower AUC (0.675) compared to other indices for the diagnosis of MASLD. WC provides a more precise assessment of visceral adiposity, while BMI serves as a useful marker of obesity that includes subcutaneous fat [26]. Fat distribution patterns are more critical than overall fat mass, with elevated abdominal visceral adiposity being more closely linked to IR and metabolic disturbances, such as hepatic steatosis, than subcutaneous fat [27]. Recognizing the substantial contribution of obesity and overweight to the progression of IR and MASLD, we found that incorporating anthropometric parameters such as WC and BMI into TyG resulted in TyG–WC (AUC: 0.865, 0.820, and 0.826 for males, females, and all, respectively) and TyG–BMI (AUC: 0.865, 0.800, and 0.820 for males, females, and all, respectively) achieving the highest diagnostic accuracy for MASLD in both genders. The findings imply that integrating TyG with BMI and WC offers a more robust evaluation, improving the effectiveness of early detection and diagnostic assessment for MASLD. In research performed by Parra BA et al. involving 161 Mexican patients, TyG–WC was identified as the most effective biomarker for diagnosing MASLD among 16 evaluated biomarkers [28]. Similar to their findings, our study identified TyG–WC as a superior diagnostic marker for MASLD, with an AUC value aligning closely with their reported 0.84. According to a study by Xue et al. in South Korea involving 1727 individuals, different TyG indices were assessed for MAFLD and NAFLD. The study reported strong diagnostic performance for TyG–WC and TyG–BMI, with AUC values of 0.81 and 0.80, respectively, closely mirroring our observed values of 0.82 for both indices. Additionally, their proposed cutoff values, 822.3 for TyG–WC and 237.7 for TyG–BMI, were very similar to our cutoffs of 826.8 and 232.7 [29]. In a study by Yu et al. involving 2605 patients, TyG–WC achieved the highest AUC (0.873) for MAFLD detection, with an optimal cutoff value of 716.743, yielding a sensitivity of 88.7% and a specificity of 71.4% [14]. A study by Yang et al. analyzing data from 71,299 participants in China reported AUC values of 0.83 for TyG, 0.92 for TyG–BMI, and 0.90 for TyG–WC when diagnosing MAFLD through an ROC curve analysis [30]. Our results align with those of prior research demonstrating the superior diagnostic value of TyG-related indices, particularly TyG–WC and TyG–BMI, in accurately identifying MASLD and MAFLD across various populations. The results underscore the applicability of TyG–WC and TyG–BMI in MASLD screening, supporting their use across diverse populations.

Parameters related to TyG have proven to be superior indicators of IR compared with markers of adipokines or visceral obesity, aligning with our findings that highlight the central role of IR in the development and progression of MASLD [31]. Previous studies have also highlighted the strong diagnostic capabilities of FLI for MASLD and NAFLD. FLI, developed by Bedogni et al., serves as a diagnostic algorithm for hepatic steatosis. This index, ranging from 0 to 100, has been validated against ultrasound-based qualitative diagnosis of fatty liver, with a threshold of ≥60 indicating a probable presence of steatosis and that of <30 effectively excluding its presence [32]. In our study, FLI demonstrated the ability to not only detect hepatic steatosis but also to diagnose MASLD with high sensitivity and specificity. The most plausible explanation for this is the inclusion of BMI, an MASLD diagnostic criterion, and WC, a crucial marker of visceral fat strongly associated with hepatic steatosis, in the calculation of FLI. A study by Song et al. involving 24,154 Korean participants compared TyG–WC, TyG–BMI, and FLI for diagnosing NAFLD and found no significant differences in AUC values (0.832, 0.827, and 0.835, respectively), aligning with our results [21]. Our study extends these findings to MASLD, emphasizing that similar outcomes were observed despite population-specific parameters. Studies evaluating FLI levels for the diagnosis of MASLD are quite limited. In a study by Thomson et al. conducted in Southern India with 65 MASLD patients, FLI was identified as a diagnostic marker for MASLD with a cutoff value of 42, yielding 63% sensitivity and specificity [33]. In a study by Crudele et al. conducted in Italy with 1069 patients, simple non-invasive tests were compared to diagnose MASLD, and FLI, with a cutoff value of 44 (AUC: 0.82; sensitivity: 74%; specificity: 75%), was identified as the most effective diagnostic test [34]. These results are closely aligned with our identified cutoff value of 41.21 (AUC: 0.83; sensitivity: 78.5%; specificity: 74%). Building on previous studies, our findings reveal that FLI, commonly used for detecting liver steatosis, can also be applied to MASLD diagnosis, highlighting the need to reassess its cutoff values.

In this study, FLI, TyG–WC, and TyG–BMI exhibited the highest AUC values for MASLD diagnosis across both sexes. Notably, variations in AUC values between genders, coupled with inconsistencies in biomarker sensitivity and specificity, underscore the pivotal role of biological sex in determining marker accuracy. These results highlight the necessity of incorporating gender as a key factor in the assessment of metabolic disorders. For example, previous studies indicate that men are prone to higher WC values due to increased visceral fat accumulation, whereas hormonal differences in women appear to exert a significant influence on BMI [35]. The identification of distinct cutoff thresholds for males and females in our study further emphasizes the need to refine diagnostic strategies and establish gender-specific reference standards. The observed gender-related discrepancies in these indices likely arise from a combination of sex-specific hormonal influences, divergent fat distribution patterns, metabolic rate variability, genetic predispositions, dietary preferences, and activity levels [35,36,37]. These findings reinforce the imperative of integrating gender-responsive and population-tailored evaluations into the creation of personalized medical approaches, ensuring more precise and effective healthcare strategies.

Our study serves as a key contribution to the evaluation of non-invasive biomarkers for MASLD diagnosis in the Turkish population. This study is strengthened by its robust sample size, meticulous screening methods, and utilization of up-to-date standardized diagnostic criteria for MASLD. Nevertheless, this study has certain limitations that warrant careful attention and acknowledgment. Firstly, the research was conducted in a single-center setting with a predominantly homogeneous ethnic group, potentially limiting the generalizability of the findings. Moreover, the detection of liver steatosis in our study is based on ultrasound imaging rather than histopathological examination of liver tissue. While ultrasound remains a prevalent non-invasive diagnostic tool, the sensitivity of conventional grayscale ultrasound is notably constrained when it comes to detecting mild degrees of hepatic steatosis. Future studies incorporating histopathological confirmation and larger, ethnically diverse populations are warranted to validate our findings. Despite its potential limitations, our study stands out as it demonstrates that TyG–WC and TyG–BMI have diagnostic value for MASLD comparable to that of FLI, a well-established diagnostic marker for MASLD. Although FLI is a well-established tool, its relatively complex calculation and potential for errors may limit its widespread application. In contrast, TyG–WC and TyG–BMI offer a simpler and more feasible alternative, particularly in low-resource settings where computational limitations may hinder the routine use of FLI.

## 5. Conclusions

Given the complexity of MASLD, which requires evidence of hepatic steatosis along with at least one cardiometabolic criterion, these indices hold significant promise for clinical practice and large-scale research applications. Moreover, their simple calculation, particularly in the case of TyG–WC and TyG–BMI, facilitates their integration into routine MASLD screening protocols, potentially enhancing early detection, especially in resource-limited settings where access to advanced diagnostic tools is restricted.

## Figures and Tables

**Table 1:** Formulas of anthropometric, biochemical–anthropometric, and non-invasive steatosis indices.

Indices	Formula
BMI	Weight(kg)/(Height(m))^2^
WHR	WC(cm)/HC(cm)
WHtR	WC(cm)/Height(cm)
BRI	364.2 − 365.5 × [1−((WC/2π)/(0.5 × height(m)))^2^]^0.5^
CI	0.109^−1^ × WC(m) × (weight(kg)/height(m))^0.5^
ABSI	WC(m)/[BMI^2/3^ × (height (m))^0.5^]
VAI (men)	(WC(cm)/(39.68 + (1.88 × BMI)) × (TG(mmol/L)/1.03))/(HDL-C(mmol/L)/1.31)
VAI (women)	(WC(cm)/(36.58 + (1.89 × BMI)) × (TG(mmol/L)/0.81))/(HDL(mmol/L)/1.52)
AVI	(2 × WC(cm) + 0.7 × (WC − HC)^2^(cm))/1000
LAP (men)	(WC(cm) − 65) × TG (mmol/L)
LAP (women)	(WC(cm) − 58) × TG (mmol/L)
TyG	Ln [(TG (mg/dL) × FPG (mg/dL))/2]
TyG–WC	TyG × WC(cm)
TyG–BMI	TyG × BMI
FLI	[(e^0.953 × ln(TG(mg/dL))+0.139 × BMI+0.718 × ln(GGT(U/L)) + 0.053 × WC(cm) − 15.745^)/(1 + e^0.953 × ln(TG(mg/dL)) + 0.139 × BMI + 0.718 × ln(GGT(U/L)) + 0.053×WC(cm) − 15.745^)] × 100
HSI	8 × (AST(U/L)/ALT(U/L)) + BMI + (+2 if female) + (+2 if diabetes present)

BMI, body mass index; WHR, waist–hip ratio; WC, waist circumference; HC, hip circumference; WHtR, waist–height ratio; BRI, body roundness index; CI, conicity index; ABSI, a body shape index; BMI, body mass index; VAI, visceral adiposity index; TG, triglyceride; HDL-C, high-density lipoprotein cholesterol; AVI, abdominal volume index; LAP, lipid accumulation product; TyG, triglyceride–glucose index; FPG, fasting plasma glucose; FLI, fatty liver index; GGT, gamma-glutamyl transferase; HSI, hepatic steatosis index; AST, aspartate transaminase; ALT, alanine aminotransferase.

**Table 2:** Characteristics of subjects with MASLD stratified by sex.

	Male (*n* = 181)			Female (*n* = 397)		
Variables	Non-MASLD (*n* = 78)	MASLD (*n* = 103)	*p*-Value	Non-MASLD (*n* = 137)	MASLD (n = 260)	*p*-Value
Age (years)	59 (45–66)	60 (56–65)	0.143	58 (51.5–65.5)	58 (55–62.75)	0.184
Smoking	33 (42.3)	43 (41.7)	0.940	25 (18.2)	53 (20.4)	0.611
Education			0.836			0.800
<High school	37 (47.4)	53 (51.5)		66 (48.2)	121 (46.5)	
High school	21 (26.9)	27 (26.2)		43 (31.4)	90 (34.6)	
>High school	20 (25.6)	23 (22.3)		28 (20.4)	49 (18.8)	
Physical activity			0.181			0.158
None	52 (66.7)	78 (75.7)		88 (64.2)	191 (73.5)	
1–2 day/week, >30 min	18 (23.1)	13 (12.6)		20 (24.6)	27 (10.4)	
3–4 day/week, >30 min	8 (10.3)	12 (11.7)		29 (21.2)	42 (16.2)	
Weight (kg)	73.63 ± 11.48	90.17 ± 13.32	<0.001	69.29 ± 11.95	81.30 ± 14.37	<0.001
BMI (kg/m^2^)	24.01 ± 2.96	29.17 ± 3.90	<0.001	26.36 ± 4.10	31.00 ± 4.76	<0.001
WC (cm)	88.06 ± 7.95	102.14 ± 10.49	<0.001	83.97 ± 10.11	97.04 ± 12.08	<0.001
HC (cm)	92.94 ± 8.33	102.01 ± 9.46	<0.001	99.05 ± 10.26	105.87 ± 11.85	<0.001
SBP (mmHg)	129.97 ± 16.85	135.59 ± 21.11	0.026	129.01 ± 18.07	131.45 ± 16.78	0.180
DBP (mmHg)	78.85 ± 11.83	82.93 ± 14.27	0.043	78.44 ± 12.17	81.00 ± 11.02	0.034
FPG (mg/dL)	90 (85–119)	104 (90–140)	<0.001	89 (82–98)	96 (89–126)	<0.001
AST (U/L)	17.50 (15.75–22)	22 (18–27)	<0.001	18 (16–22)	21 (18–25)	<0.001
ALT (U/L)	17 (12.75–21.25)	22 (16–32)	<0.001	16 (13–21)	20 (16–25)	<0.001
GGT (U/L)	21 (17–31)	25 (17–39)	0.154	18 (14.30–24)	22 (17–30)	<0.001
TG (mg/dL)	112 (81–133)	145 (96–178)	<0.001	114 (94–141)	145 (104–191)	<0.001
HDL-C (mg/dL)	46.50 (41–56)	44 (40–48)	0.005	51 (45–55)	45 (42–50)	<0.001
WHR	0.94 ± 0.05	1.00 ± 0.07	<0.001	0.84 ± 0.07	0.91 ± 0.08	<0.001
WHtR	0.50 ± 0.05	0.58 ±0.06	<0.001	0.51 ± 0.06	0.60 ± 0.07	<0.001
BRI	3.49 ± 0.97	5.11 ± 1.45	<0.001	3.81 ± 1.36	5.56 ± 1.75	<0.001
CI	1.25 ± 0.08	1.31 ± 0.09	<0.001	1.18 ± 0.09	1.25 ± 0.10	<0.001
ABSI	0.080 ± 0.005	0.081 ± 0.005	0.172	0.074 ± 0.006	0.077 ± 0.006	<0.001
VAI	1.33 (0.93–1.67)	1.87 (1.25–2.85)	<0.001	1.77 (1.44–2.29)	2.60 (1.89–3.54)	<0.001
AVI	15.67 ± 2.82	21.12 ± 4.28	<0.001	14.50 ± 3.44	19.23 ± 4.62	<0.001
LAP	27.8 (19.7–37)	58.8 (35.2–82.5)	<0.001	30.5 (21.5–48)	62.2 (40.2–88.4)	<0.001
TyG	8.59 ± 0.47	8.91 ± 0.62	<0.001	8.58 ±0.40	8.92 ± 0.56	<0.001
TyG–WC	757.1 ± 79.7	911.5 ± 120.8	<0.001	721.9 ±98.6	866.7 ± 124.8	<0.001
TyG–BMI	206.57 ± 29.75	260.53 ± 41.67	<0.001	266.44 ± 36.83	296.9 ± 47.26	<0.001
FLI	30.29 ± 17.22	63.31 ± 23.45	<0.001	31.66 ± 21.56	62.77 ± 25.61	<0.001
HSI	32.10 (29.75–35.26)	37.86 (35.36–41.72)	<0.001	33.52 (30.95–37.57)	39.33 (35.72–47.57)	<0.001

Data are presented as mean ± SD or median (25–75% inter-quartiles), while *p*-values are calculated by Pearson’s chi-squared test and Student’s *t*-test or Mann–Whitney U test. Statistically significant values are marked in bold. BMI, body mass index; WC, waist circumference; HC, hip circumference; SBP, systolic blood pressure; DBP, diastolic blood pressure; FPG, fasting plasma glucose; AST, aspartate transaminase; ALT, alanine aminotransferase; GGT, gamma-glutamyl transferase; TG, triglycerides; HDL-C, high-density lipoprotein cholesterol; WHR, waist–hip ratio; WHtR, waist–height ratio; BRI, body roundness index; CI, conicity index; ABSI, a body shape index; VAI, visceral adiposity index; AVI, abdominal volume index; LAP, lipid accumulation product; TyG, triglyceride–glucose index; FLI, fatty liver index; HSI, hepatic steatosis index.

**Table 3:** Comparisons between men and women with MASLD (*n*= 363).

Criteria	Men (*n* = 103)	Women (*n* = 260)	*p*-Value
Diabetes mellitus	57 (55.3%)	129 (49.6%)	ns
Hypertension	63 (61.2%)	180 (69.2%)	ns
Hyperglycemia criterion	55 (53.4%)	112 (43.1%)	ns
HDL-cholesterol criterion	32 (31.1%)	167 (64.2%)	<0.001
Triglyceride criterion	44 (42.7%)	115 (44.2%)	ns
WC criterion	80 (77.7%)	239 (91.9%)	<0.001
BMI criterion	90 (87.4%)	233 (89.6%)	ns

The data are presented as numbers (%), while *p*-values are calculated by Pearson’s chi-squared test. Statistically significant values are marked in bold. An FPG > 126 mg/dL or ongoing anti-diabetic treatment indicated type 2 diabetes diagnosis. Systolic arterial blood pressure (SAP) ≥ 130 mmHg, diastolic arterial blood pressure (DAP) ≥ 85 mmHg, and/or treatment with antihypertensive agents indicated hypertension. An FPG ≥ 100 indicated hyperglycemia. Waist circumference was considered pathological above 80 cm in women and 94 cm in men. To characterize dyslipidemia, HDL cut offs of <40 mg/dL in males and <50 mg/dL in females and triglyceride values ≥ 150 mg/dL for both genders were considered pathological. BMI, body mass index; ns, not significant.

**Table 4:** ROC curve analysis and pairwise comparison of the AUCs for each variable for diagnosing MASLD.

Parameter	AUC	Cutoff	Sensitivity	Specificity	95% Cl	*p*-Value
BMI	0.792	26.40	79.1%	68.8%	0.794–0.830	<0.001
WHR	0.671	0.8608	82.6%	43.3%	0.626–0.716	<0.001
WHtR	0.803	0.5327	80.7%	67.4%	0.767–0.840	<0.001
BRI	0.803	4.1441	78.0%	70.2%	0.767–0.840	<0.001
CI	0.687	1.2407	62.8%	65.6%	0.642–0.732	<0.001
ABSI	0.586	0.0778	54.8%	62.3%	0.538–0.635	<0.001
AVI	0.805	17.68	69.4%	80.9%	0.770–0.841	<0.001
TyG	0.675	8.924	46.8%	80.5%	0.631–0.718	<0.001
TyG–WC	0.826	826.88	70.2%	83.3%	0.792–0.860	<0.001
TyG–BMI	0.820	232.75	78.8%	74.0%	0.785–0.855	<0.001
FLI	0.830	41.21	78.5%	74.0%	0.797–0.863	<0.001
HSI	0.783	35.02	80.7%	67.0%	0.743–0.822	<0.001
**Pairwise Comparison**		**Difference AUC**		**95% CI**		***p*-Value**
WHtR vs. AVI		0.002		−0.015–0.019		0.819
TyG–WC vs. TyG–BMI		0.006		−0.020–0.032		0.664
FLI vs. TyG–WC		0.004		−0.013–0.021		0.643
FLI vs. TyG–BMI		0.010		−0.008–0.028		0.283
AVI vs. TyG–WC		0.020		−0.003–0.036		0.015
FLI vs. AVI		0.025		0.003–0.045		0.022
TyG–WC vs. BRI		0.022		0.000–0.043		0.041

*p*-values for the parameters we recalculated using ROC analysis, and statistical comparisons between the parameters were performed using the DeLong test. Statistically significant values are marked in bold. AUC, area under curve; CI, confidence interval; BMI, body mass index; WHR, waist–hip ratio; WHtR, waist–height ratio; BRI, body roundness index; CI, conicity index; ABSI, a body shape index; AVI, abdominal volume index; TyG, triglyceride–glucose index; WC, waist circumference; FLI, fatty liver index; HSI, hepatic steatosis index.

**Table 5:** The cutoff, sensitivities, specificities, and area under the curve of different variables for the screening of MASLD in men and women.

Sex	Parameter	AUC	Cutoff	Sensitivity	Specificity	*p*-Value
Men	BMI	0.838	26.40	71.8%	84.6%	<0.001
	WHR	0.707	0.9955	55.3%	76.9%	<0.001
	WHtR	0.827	0.5209	83.5%	71.8%	<0.001
	BRI	0.827	3.7596	83.5%	71.8%	<0.001
	CI	0.683	1.3018	58.3%	73.1%	<0.001
	ABSI	0.551	0.0874	21.4%	92.3%	0.237
	VAI	0.695	1.6827	58.3%	76.9%	<0.001
	AVI	0.854	16.96	84.5%	79.5%	<0.001
	LAP	0.799	44.59	63.1%	88.5%	<0.001
	TyG	0.664	8.9309	49.5%	80.8%	<0.001
	TyG–WC	0.865	833.92	71.8%	88.5%	<0.001
	TyG–BMI	0.865	229.21	75.7%	84.6%	<0.001
	FLI	0.860	34.76	91.3%	71.8%	<0.001
	HSI	0.818	35.33	75.7%	76.9%	<0.001
Women	BMI	0.772	27.60	76.2%	68.6%	<0.001
	WHR	0.735	0.8653	75.8%	65.0%	<0.001
	WHtR	0.789	0.5535	74.2%	70.8%	<0.001
	BRI	0.789	4.4164	74.2%	70.8%	<0.001
	CI	0.723	1.1922	75.8%	60.6%	<0.001
	ABSI	0.638	0.0773	51.9%	73.0%	<0.001
	VAI	0.711	2.2644	61.2%	75.2%	<0.001
	AVI	0.796	25.20	81.5%	66.4%	<0.001
	LAP	0.784	38.41	79.6%	67.2%	<0.001
	TyG	0.680	8.9146	45.8%	81.0%	<0.001
	TyG–WC	0.820	777.66	78.1%	75.2%	<0.001
	TyG–BMI	0.800	232.52	81.9%	67.2%	<0.001
	FLI	0.817	41.72	75.4%	75.2%	<0.001
	HSI	0.762	34.80	82.7%	62.0%	<0.001

*p*-values for the parameters we recalculated using ROC analysis. Statistically significant values are marked in bold. AUC, area under curve; CI, confidence interval; BMI, body mass index; WHR, waist–hip ratio; WHtR, waist–height ratio; BRI, body roundness index; CI, conicity index; ABSI, a body shape index; VAI, visceral adiposity index; AVI, abdominal volume index; LAP, lipid accumulation product; TyG, triglyceride–glucose index; WC, waist circumference; FLI, fatty liver index; HSI, hepatic steatosis index.

**Table 6:** MASLD OR and 95% CI based on the TyG–WC, TyG–BMI, and FLI quartiles.

	Men		Women	
	Univariate	Multivariate *	Univariate	Multivariate *
	OR (95% CI)	Adj OR (95% CI)	OR (95% CI)	Adj OR (95% CI)
TyG–WC				
Q1	1	1	1	1
Q2	3.96 (1.46–10.78)	3.51 (1.21–10.18)	2.52 (1.41–4.51)	2.68 (1.48–4.87)
Q3	15.38 (5.43–43.54)	15.23 (5.14–45.11)	11.37 (5.74–22.53)	11.46 (5.74–22.90)
Q4	116.71 (22.84–596.25)	123.55 (23.34–653.89)	28.82 (11.98–69.36)	31.13 (12.77–75.89)
*p*-value	<0.001	<0.001	<0.001	<0.001
TyG–BMI				
Q1	1	1	1	1
Q2	2.20 (0.852–5.71)	3.80 (1.09–8.68)	3.25 (1.81–5.84)	3.36 (1.84–6.12)
Q3	16.44 (5.81–46.13)	30.14 (8.72–104.16)	8.05 (4.24–15.25)	8.33 (4.34–16.00)
Q4	41.00 (11.63–144.54)	67.49 (16.91–269.34)	43.24 (15.96–117.12)	47.13 (17.21–129.08)
*p*-value	<0.001	<0.001	<0.001	<0.001
FLI				
Q1	1	1	1	1
Q2	3.38 (1.28–8.88)	4.24 (1.51–11.92)	3.27 (1.81–5.90)	3.18 (1.75–5.79)
Q3	11.74 (4.32–31.84)	14.29 (4.99–41.70)	9.65 (5.02–18.65)	9.43 (4.87–18.23)
Q4	99.43 (19.86–497.77)	122.33 (23.26–643.38)	45.37 (16.72–123.13)	46.13 (16.90–125.94)
*p*-value	<0.001	<0.001	<0.001	<0.001

Values are presented as odds ratios (95% CI), while *p*-values are calculated by univariate and multivariate logistic regression analysis. CI, 95% confidence interval; TyG, triglyceride–glucose index; WC, waist circumference; BMI, body mass index; FLI, fatty liver index. * Adjusted for age, education, and physical activity.

## Data Availability

Data are available on request due to restrictions (e.g., privacy, legal, or ethical reasons).
